# Hôtel Dieu

**Published:** 1830-10-01

**Authors:** 


### HOTEL DIEU.

CASE OF ACUTE NEURALGIA RHEUMATICA OF THE DIAPHRAGM. By Dr. COUDRET, interne à L'Hôtel Dieu.

This case is related by the patient himself, who, as a medical man, may well be considered as capable of appreciating the nature and seat of his own malady.

Aged about 29 years, of nervous temperament, but enjoying good health, he had been subject, for some time, after exposure to cold in the amphitheatre, to slight, intercostal pain in the left side, occasionally exchanged for pains of a colicky nature in the bowels—for coryza—and for cynanche tonsillaris. On the 28th January 1830, when the temperature was 12° of Reamur below zero, he was imprudent enough to have his hair cut close, immediately after which he became affected with slight bronchitis and some inflammation of the tunica conjunctiva. On the 8th of February, these affections being still in existence, he was exposed, while warm, to a current of cold air, and thence repaired to the Hôtel Dieu, where he did not, at first, experience any particular inconvenience. At 5 o'clock in the afternoon, he dined, though not with appetite. At 8 o'clock, he experienced a febrile horripilation, general malaise, heaviness of head, pain in his joints, disinclination to motion. Soon after this a shiver was felt, the coldness being succeeded by febrile re-action, violent pain in the limbs, the loins, and head, &c. The night was very restless, the pains, though general at first, being concentrated ultimately in the left lumbar region, and at the lower part of the chest on the same side. Next day, 9th February, the skin was still dry and burning—the restlessness incessant—head-ache intense—feeling of great sanguineous congestion about the face—tongue pasty, but not red—thirst considerable—some nausea—urine pale—bowels costive—cough, with catarrhal expectoration—the pain in the loins and side increased by the act of coughing. On percussion, the chest was every where sonorous. At each effort to inspire, the patient found himself checked by a sudden and violent pain, apparently in the situation where the diaphragm is attached to the false ribs of the left side, and also to the spine. He conceived that he felt this same pain in the tendinous centre of the diaphragm, whence it appeared to radiate along the course of the left diaphragmatic nerve to the neigbourhood of the clavicle of that side. The act of turning, the least effort to breathe, to expel the urine, to eructate, or blow his nose, increased this pain to exquisite torture. His common respiration was also short and embarrassed. Lastly, he felt a sensation in his left arm similar to what is described by those who labour under angina pectoris. Nothing was felt about the right side of the chest. Careful pressure was made on all parts of the abdomen, but no uneasiness was thereby produced. These phenomena convinced the patient that the disease was not pleuritis; but that the seat of the malady was the diaphragm. The fever was now very acute, the pulse full, hard, and quick—in short, every thing indicated the necessity of venesection. His friend, who was with him in the Hotel Dieu, immediately bled him to a large amount, without producing faintness. The blood was rich, but very little inflamed. Feeling some nausea, he took several glasses of warm water, and cleared his stomach, but without bringing up any remains of food, or any bile. He now felt better, and had a mild perspiration. But the pain above-described continued, and the pyrexial symptoms were soon renewed, with discontinuance of the perspiration. Thirty-five leeches were now applied to the anus, followed by a hot poultice to the same, and also to the feet. These means completely removed the head-ache, and much of the general malaise—the perspiration was reproduced—and he would have experienced some repose, had not the pains in the region of the diaphragm continued to harrass him incessantly. He now balanced between the application of 40 or 50 leeches to the chest, or sinapisms to the same part. He determined in favour of the latter—and managed them with great dexterity, contriving to keep up a constant counter-irritation over the left side and back of the thorax, without inducing vesication. Two days of this discipline gave complete relief to his sufferings. On the 12th of February, he was free from complaint excepting debility.

The author thinks, and we are inclined to agree with him, that the phenomena which he has described, and severely felt, indicate a rheumatic affection of the diaphragm—a disease rarely delineated by medical writers, probably on account of the inability of non-medical patients to accurately ascertain the seat or kind of their own dolorous sensations.—JOURNAL COMPLÉMENTAIRE.

II. CASES ILLUSTRATIVE OF THE EFFECTS OF COLD AFFUSIONS; WITH REMARKS BY M. RECAMIER.*

A man about 35 years of age, of strong habit of body, and apparently sanguineous temperament, was brought into the Hôtel Dieu in a state of insensibility in the early part of last March. He was a juggler, and was found in the condition above-mentioned in his own apartment. At the morning's visit of M. Recamier he was delirious, but not violently so, knew when he was

* Journ. Hebdom. No. 79.

addressed by name, but was not able to answer questions. His face was injected, his hair on end, his eyes wild and haggard, his discourse incoherent. He had no stertor nor paralysis, and when his hand was raised was able to maintain it so. The abdomen free from tenderness, the pulse 75, regular but small, the skin moderately warm and dry. M. Recamier considered the affection as purely nervous, styled it nervous stupor, and prescribed affusions from head to foot of water at the temperature of 18° Reaumur. The delirium immediately ceased, and on the next day the patient was walking about the ward.

M. Recamier, at least the reporter for him, enters into a subtle and lengthy disquisition on the modus operandi of the cold affusion in this and other cases. The pith of a page or two of verbiage is merely this, that the remedy acts on the nervous system, through the medium of the shock to the surface of the body. There is no great discovery in this. The best mode of making the affusion is to procure a vessel with a large mouth, from which the water, at the temperature of 18° Reaumur, is to be poured for the space of six minutes on the head of the patient standing in the bath. The mouth and nostrils should be protected, and it might be useful to place a large wet sponge on the head, by which means the water would run down over the body and produce much the same effect as the affusion.

The reporter concludes the subject with a case of those anomalous hysterical symptoms which a bad practitioner will almost always mistake, a good one as generally recognize. Cough, slight hæmoptysis, palpitations, obstinate rejection of food, &c. were the symptoms that followed in succession or appeared in combination with each other, and obstinately resisted the antispasmodics and sedatives. The reporter, M. Dupré, then had recourse to the bath, the body being immersed in water of the temperature of 26° Reaumur, whilst affusions at 18° were directed on the head. This proceeding was adopted for 15 minutes at a time and with very good effect. All well-informed practitioners in this country are in the habit of using measures similar to the above, in nervous and hysterical disorders. The shower-bath is a less clumsy method of effecting cold affusion than the sponge or the pitcher, but under certain circumstances the latter may be more readily obtained. It is always a good precaution in commencing the use of the shower-bath, to let the patient stand with the feet in warm water, especially if she be a delicate and nervous girl. The benefit derived from this plan in chorea, and the nervous pains in the chest and abdomen of young women, is frequently considerable.

III. Syncope from Hæmorrhage.*

A woman, æt. 37, affected with organic disease of the uterus was about to have the organ extirpated by M. Recamier, when on the 7th April, she was suddenly seized with profuse uterine hæmorrhage, in consequence of which she fainted and remained for a long time insensible. She was placed on a bed, cold water dashed in her face, wetted towels laid on the abdomen, and an ether draught administered, by which means she was recovered from her dangerous syncope.

The case is detailed for the purpose of mentioning M. Recamier's practice, and relating his clinique on the occasion. The patient should be immediately placed in the recumbent posture, and cold water dashed, not pleno rivo but in drops, upon her face, whilst frictions may be made in the region of the heart. If the syncope depend on uterine hæmorrhage, cloths wetted with cold water should be applied to the abdomen, and bladders filled with pounded ice to the vulva and hypogastrium. M. Recamier does not approve of plugging the vagina, as it only prevents the blood escaping, nor of injections as they tend to detach the coagula and renew the hæmorrhage if it is already ceasing. M. Recamier prefers acting on the capillaries of the bleeding organ by applying refrigerants to those parts or organs with which it is sympathetically

* Journ. Hebd. No. 83.

connected. Thus cold drinks are useful, the nitric and sulphuric acids with æther are also extremely serviceable, and so are ether draughts. M. Recamier approves of employing moral agents on the nervous system, because as they often give rise to hæmorrhagies in the nervous ladies of the present day, they also conspire to arrest them. So much for M. Recamier's lecture on the treatment of syncope.

IV. DISSECTION OF A RECENT DISLOCATION OF THE HEAD OF THE HUMERUS.*

A man was seized with apoplexy, and in falling to the ground dislocated the upper extremity of the humerus. He was brought into the Hôtel Dieu and died shortly afterwards, when the extremity was carefully dissected, and exhibited to the pupils by M. Dupuytren at his Clinique, on the 12th of February last. The capsule was torn at its inferior part very near its insertion into the neck of the humerus. The head passed freely out, and could be readily returned within it; the greater tuberosity was fractured and detached from the rest of the bone. In lecturing on this piece of surgical anatomy, M. Dupuytren particularly alluded to the facility with which the head of the bone could be made to pass and repass the rent in the capsule. Dessault and many surgeons of the present day have entertained the opinion, that the small size of the laceration in the capsule presents the greatest obstacle to the reduction of the dislocation; and Dessault carried this notion so far, that he insisted on and practised extended motions of the extremity to enlarge the aperture. This idea appears to M. Dupuytren to be much less generally correct than its advocates suppose; but he does not deny the occasional occurrence of difficulties from this cause. We are not informed whether M. Dupuytren's opinion is grounded on this, or more extensive observations; if the former, we need scarcely observe that a solitary fact proves little, though probably this able surgeon is correct.

V. CONGENITAL DISLOCATION OF THE HEAD OF EACH RADIUS.

In February, 1830, a curious piece of pathology was shewn in the amphitheatre of the Hôtel Dieu. The dissection was made by M. Loir, interne of the hospital and prosector to M. Dupuytren. The upper extremity of either radius was displaced from its natural situation, and was situated behind the inferior extremity of the humerus, above which it mounted an inch at the least. M. Dupuytren met with a similar dislocation of the radius twenty or twenty-five years ago, but he cannot distinctly remember whether it was congenital or existed on both sides. In the present instance, it would be difficult to determine precisely whether the dislocation of each radius was congenital, or produced by some violent twisting inwards of both fore-arms, or was "the result of a white swelling."

VI. FOREIGN BODY IN THE TRACHEA—TRACHEOTOMY.

A little girl, eight years of age, accidentally swallowed a bean, which passed into the trachea and there remained. A violent suffocative cough ensued, and some medical men who were summoned in haste exhibited an emetic without avail. The remainder of the day was passed in alternations of quiet and paroxysms of suffocation, and on the following evening, Feb. 12th, she was admitted into the Hôtel Dieu. She suffered during the night from violent attacks of dyspnœa, and had several alarming fits of syncope. In the morning of the 13th the cough was still most distressing, and on applying the ear to the trachea the foreign body could be heard agitated within it. M. Dupuytren determined on tracheotomy, which he performed in the following manner. The child being placed on a bed and held by assistants, with the head thrown backwards, an incision nearly an inch and a quarter in length was made along the median line, a little above the superior border of the sternum. The skin and subjacent cellular

* This and the following articles from the Journ. Hebd. No. 80.

tissue were divided, and the muscles separated from each other, when the trachea was exposed to view. M. Dupuytren, in the next place, with a straight-pointed bistoury guided on the nail of the left fore-finger, divided from above downwards several of the cartilaginous rings of the trachea, with the ligamentous structure uniting them. No air escaped at first, but the edges of the wound being held apart, and the head slightly bent, it issued with much force, and after some violent efforts at inspiration, the bean was driven out from the wound, and fell on the breast of the little patient. The respiration then became natural, the cough ceased, and the child appeared to be well. The edges of the wound were carefully cleansed from the blood, some simple dressing placed in front of the neck, with one or two gentle compresses, and a moderately tight bandage over all. The bean was five lines in length, three in breadth, and as many in thickness.

In the evening there was much fever, and venesection was required. Next day the respiration was entirely carried on by the tracheal opening, and some pain was experienced in the larynx. Leeches and diluents were employed with great advantage, and on the 15th the breathing was free and unembarrassed, and only a little whistling through the wound was heard. On the 20th, the edges of the wound were brought together, and some days afterwards, its extent being reduced to one-third its former size, the child was taken home by her parents. In a month after the operation they brought her back, and there still remained a minute aperture through which issued a stream of air.

A clinical lecture on the above case was delivered by M. Dupuytren. In order to make the first incision through the skin and subcutaneous cellular tissue, the head should be directed backwards, but when the incisions are completed, this position of the head is very unfavourable for the free escape of air or of the foreign body. In the foregoing case the air was prevented from escaping so long as the head was kept backwards, and the bean was expelled from the larynx when the neck was bent forwards. One caution is requisite in the performance of the operation; between the muscles in front of the trachea is a cellular interspace, which, when divided, forms a kind of cavity, and in this the operator may get perplexed, if not aware of its exact nature. M. Dupuytren particularly directed the attention of his hearers to the shock communicated by the foreign body to the parietes of the trachea, and distinguished by the ear or even by the hand. It was present in this instance, but is not perceived in all cases, nor even at all periods of the same case. If adherent, of course the foreign body cannot be moved, and if not moveable, it obviously is incapable of communicating an impulse to the parietes of the trachea. In dressing the patient after the operation, M. Dupuytren is careful at first not to bring the edges of the wound together, in order to prevent any hazard of emphysema. At the end of some days, however, when the cellular tissue is in some degree condensed by inflammation, and no danger of emphysema exists, there can be no objection to approximating the sides of the opening. No lint should be placed over the wound, as it has occasionally penetrated into the trachea, and induced dangerous consequences.

### VI. Foreign Body in the Larynx—Extraction.

A female between 45 and 50 years of age, presented herself at the Hôtel Dieu on the 20th March, 1830, with violent pain at the top of the larynx on swallowing, or even in the act of respiration. She stated that six or seven days previously she had swallowed a fish bone, since which she had experienced the distressing sensation in question, and that latterly it had been more severe than at first. The mouth being widely opened, M. Dupuytren introduced his finger as far as the epiglottis, which he thus examined; near its base and posteriorly he could feel a substance projecting like a pin's head, but the rest of it was concealed beneath the epiglottis and could not be examined. M. Dupuytren, maintaining his fore-finger on the foreign body, introduced by its guidance a pair of ring-forceps, seized the fish-bone, for such it proved to be, and gently extracted it; it was about an inch and a quarter in length. The operation was attended with little pain and no hæmorrhage, and the woman probably required no further assistance, for she never returned to the hospital.

